# Discriminative analysis of non-linear brain connectivity in schizophrenia: an fMRI Study

**DOI:** 10.3389/fnhum.2013.00702

**Published:** 2013-10-22

**Authors:** Longfei Su, Lubin Wang, Hui Shen, Guiyu Feng, Dewen Hu

**Affiliations:** ^1^College of Mechatronics and Automation, National University of Defense TechnologyChangsha, China; ^2^Department of Automatic Control, College of Mechatronics and Automation, National University of Defense TechnologyChangsha, China; ^3^Department of Automation, Institute of Computing Technology, Beijing Jiaotong UniversityBeijing, China

**Keywords:** schizophrenia, resting-state functional connectivity, non-linear, extended maximal information coefficient, compensatory

## Abstract

**Background**: Dysfunctional integration of distributed brain networks is believed to be the cause of schizophrenia, and resting-state functional connectivity analyses of schizophrenia have attracted considerable attention in recent years. Unfortunately, existing functional connectivity analyses of schizophrenia have been mostly limited to linear associations.

**Objective**: The objective of the present study is to evaluate the discriminative power of non-linear functional connectivity and identify its changes in schizophrenia.

**Method**: A novel measure utilizing the extended maximal information coefficient was introduced to construct non-linear functional connectivity. In conjunction with multivariate pattern analysis, the new functional connectivity successfully discriminated schizophrenic patients from healthy controls with relative higher accuracy rate than the linear measure.

**Result**: We found that the strength of the identified non-linear functional connections involved in the classification increased in patients with schizophrenia, which was opposed to its linear counterpart. Further functional network analysis revealed that the changes of the non-linear and linear connectivity have similar but not completely the same spatial distribution in human brain.

**Conclusion**: The classification results suggest that the non-linear functional connectivity provided useful discriminative power in diagnosis of schizophrenia, and the inverse but similar spatial distributed changes between the non-linear and linear measure may indicate the underlying compensatory mechanism and the complex neuronal synchronization underlying the symptom of schizophrenia.

## Introduction

Schizophrenia, which is characterized by delusions, auditory hallucinations, and impairments in memory, attention, and executive function, is one of the most devastating, cryptic, and costly psychiatric disorders (van Os et al., 2010; Sui et al., 2012). It brings not only great suffering to patients but also significant costs to society. Traditionally, the diagnosis of schizophrenia depends on the observation of psychiatric symptoms and longitudinal courses, while modern diagnoses of such psychiatric disorders require objective neurological measures (Kendler, 2009; Insel et al., 2010; Shen et al., 2010). The underlying etiology and mechanisms of schizophrenia are still unclear, but it is believed that the dysfunctional integration of distributed brain networks leads to this mental disease (Friston and Frith, 1995; Andreasen et al., 1998). Thus, based on fMRI data, functional connectivity research on schizophrenia has attracted considerable attention in recent years (Camchong et al., 2011; Pettersson-Yeo et al., 2011; Bassett et al., 2012; Fornito et al., 2012; Liu et al., 2012; Tu et al., 2012; Vertes et al., 2012; Zalesky et al., 2012). Furthermore, the introduction of multivariate pattern classification to behavioral and cognitive neuroscience increases the potential of functional connectivity in clinical diagnoses of this mental disease (Shen et al., 2010; Fan et al., 2011).

Functional connectivity, defined as statistical associations between remote neurophysiological events, aims to characterize the communication between different brain regions (Friston et al., 1993). Most functional connectivity analyses use temporal correlations or covariance to examine the simultaneous coupling between two time series. Fluctuations in the blood oxygen level dependence (BOLD) signal have attracted attention since the 1990's (Biswal et al., 1995, 1996). In contrast to functional connectivity, which need previous acknowledge about the disease and did not estimate the potential functional connectivity of other ROIs (Lowe et al., 1998, 2000; Xiong et al., 1999; Cordes et al., 2000; Hampson et al., 2002), whole brain functional connectivity analyses of schizophrenia which require less field specific knowledge have attracted considerable attention in recent years (Liang et al., 2006; Liu et al., 2006; Lynall et al., 2010; Alexander-Bloch et al., 2013).

Previous whole brain functional connectivity analyses of schizophrenia which primarily used temporal correlation or covariance as the measure limited to consideration of only the linear associations (Liang et al., 2006; Liu et al., 2006; Lynall et al., 2010; Alexander-Bloch et al., 2013). Because correlation calculations in the full-lag space are computationally expensive (Cecchi et al., 2007), correlation related studies have prevalently used the zero*th* lag correlation. The hemodynamic response can significantly reduce the computational expense for its limited duration (Li et al., 2009), but presents another problem, namely, the hemodynamic response function (HRF) varies across subjects and brain regions (Buckner et al., 1998; Miezin et al., 2000; Lee et al., 2001; Saad et al., 2001), and the zero*th* lag correlation is sensitive to changes in regional HRFs. Specifically, the simple correlation (zero*th* lag) is sensitive to the lag between time series that cannot sufficiently depict the functional interactions of the human brain (Smith et al., 2011). Thus, we believe that the underlying neural activity cannot be accurately reflected by the zero*th* lag correlation- or covariance-based methods.

Investigating functional connectivity in schizophrenia from the frequency domain is an alternative of the temporal correlation (Fallani et al., 2010; Salvador et al., 2010). Although the spectral analogs of functional connectivity such as coherence fixed some of the problems (i.e., lag between time series) faced by the simple correlation methods, coherence explores only linear relationships between time series (Sun et al., 2004; Smith et al., 2011). However, the non-linearity of the HRF has been reported by several studies (Buxton et al., 1998; Friston et al., 2003; de Zwart et al., 2009; Daunizeau et al., 2012). As an output of HRF, the BOLD signal also has non-linear properties (Vazquez and Noll, 1998; Xie et al., 2008a,b; Zhang et al., 2008). Notably, non-linear relationships between time series extracted from resting state BOLD signals have also been confirmed (Lahaye et al., 2003).

The first goal of this study is to evaluate the discriminative power of non-linear functional connectivity in schizophrenia which may have potential application in diagnosis of neuropsychiatric disorders. The second goal is to investigate the changes of the non-linear functional connectivity in schizophrenia which may contribute to the pathophysiology of schizophrenia. Aiming at exploring non-linear associations in schizophrenia, we present a novel method that introduces the extended maximal information coefficient (eMIC) to construct functional connectivity of the whole brain.

The eMIC represents the difference between the MIC which is a statistical method for detecting various associations between pairs of variables in large data sets (Reshef et al., 2011) and the square of the Pearson correlation coefficient (PCC). In a previous study, the square of PCC was proved to be equal to the Hilbert-Schmidt Independence Criterion (HSIC) and gave excellent performance in feature selection (Song et al., 2007). By applying the MIC, PCC and eMIC with fMRI data, we investigated the discriminative power of non-linear and linear functional connectivity. Based on the classification result, we further evaluated the changes and spatial distribution of linear and non-linear functional connectivity in schizophrenia.

## Materials and methods

### Materials

#### Participants

The fMRI data used in this study were acquired from 64 participants who were all right-handed native Chinese speakers. Participants consisted of 32 patients suffering from schizophrenia and 32 healthy controls. All the patients were recruited from outpatient departments and inpatient units at the Department of Psychiatry, Second Xiangya Hospital of Central South University in Changsha, China, between March 2006 and October 2007, and satisfied the Diagnostic and Statistical Manual of Mental Disorders, Fourth Edition (DSM-IV; American Psychiatric Association, 1994) criteria. Five patients were medication-free, while the others accepted antipsychotic drugs at the time of image acquisition. The healthy subjects were recruited by advertisements, and matched to the patients on age and gender. None of them had major head trauma, history of alcohol or drug dependence, or history of neurological disorder. Written informed consents were obtained from all the subjects who took part in this study. This study was approved by the Medical Research Ethics Committee of the Second Xiangya Hospital, Central South University. Details about the participants were displayed in Table 1.

**Table 1:** **Characteristics of participants in this study**.

**Variables**	**Mean + *SD***		***P*-value**
	**Patient**	**Control**	
Sample size	32	32	
Gender(M/F)	25/7	23/9	0.85
Age(years)	24 ± 5.66	25.01 ± 4.50	0.92
PANSS	80.06 ± 16.55		

PANSS: Positive and Negative Syndrome Scale

#### Imaging protocol

In the experiments, subjects were instructed simply to keep their eyes closed, to relax, remain awake and perform no specific cognitive exercise. After each session, subjects were asked whether they were awake in the previous session and all the subjects confirmed. MRI scans were performed with a 1.5T GE Signa System (GE Signa, Milwaukee, Wisconsin, USA). To reduce the head movements, subjects' heads were fixed by using foam pads with a standard birdcage head coil. The functional MRI images were collected by using a gradient-echo echo planar imaging sequence. The imaging parameters were as follows: repetition time/echo time = 2000/40 ms, thickness/gap = 5/1 mm, field of view = 240 × 240 cm, flip angle = 90°, matrix = 64 × 64, and slices = 20. Each functional resting state session lasted ~6 min, and 180 volumes were obtained.

#### Data preprocessing

Data preprocessing was performed by using statistical parametric mapping software package (SPM2, Wellcome Department of Cognitive Neurology, Institute of Neurology, London, UK, http://www.fil.ion.ucl.ac.uk/spm/). In each subject, the first 5 volumes of the scanned data were discarded for magnetic saturation effects. The remaining 175 volumes were corrected by registering and reslicing for head motion. Next, the volumes were normalized to the standard echo planar imaging template in the Montreal Neurological Institute (MNI) space. The resulting images were spatially smoothed with a Gaussian filter of 8 mm full-width half-maximum kernel, detrended to abandon linear trend and then temporally filtered with a Chebyshev band-pass filter (0.01–0.08 Hz). The registered fMRI volumes with the MNI template were further divided into 116 regions according to the automatic anatomical labeling atlas (Schmahmann et al., 1999; Tzourio-Mazoyer et al., 2002). The atlas divides the cerebrum into 90 regions (45 in each hemisphere) and divides the cerebellum into 26 regions (nine in each cerebellar hemisphere and eight in the vermis). All region of interest masks were generated using the free software WFU_PickAtlas (version 3.0, http://www.ansir.wfubmc.edu) (Maldjian et al., 2003).

Regional mean time series were obtained for each individual by averaging the functional MRI time series over all voxels in each of the 116 regions. Each regional mean time series was further corrected for the effects of head movement by regression on the time series of translations and rotations of the head estimated in the course of initial movement correction by image realignment. The residuals of these regressions constituted the set of regional mean time series used for functional connectivity analysis (Achard et al., 2006).

We evaluated functional connectivity between each pair of regions using the MIC, PCC, and eMIC. Thus, for each subject, we obtained three resting state functional networks captured by three 116 × 116 symmetric matrixes respectively. Removing 116 diagonal elements, we extracted the upper triangle elements of the functional connectivity matrix as classification features, i.e., the feature space for classification was spanned by the (116 × 115)/2 = 6670 dimensional feature vectors.

### Methods

#### MIC and eMIC

In this section, we provide a brief description of the MIC and eMIC for detecting the association between two time series. Two time series can be viewed as a set of ordered data pairs. The MIC between a set of ordered pairs will not change if the rank of pairs is disturbed but the relative ranks of the *x*- and *y*-values do not change. If two variables are related to each other, then a grid can be drawn on the scatterplot of the two variables that encapsulates that relationship. Based on this concept, this method investigated all the grids up to the maximal grid resolution, which depends on the sample size (Reshef et al., 2011). The MI is defined as follows:

(1) $$\begin{array}{l} MI(X;Y)=H(X)-H(X|Y)\\ \;=H(Y)-H(Y|X)\\ \;=H(X)+H(Y)-H(X,Y) \end{array}$$

where *H*(*X*) and *H*(*Y*) are the marginal entropies, *H*(*Y*|*X*.) and *H*(*Y*|*X*.) are the conditional entropies, and *H*(*X, Y*) is the joint entropy of *X* and *Y*.

Formally, let *G*_*x* × *y*_ be all the possible partitions with rows *x* and *y* columns (width of rows are different; width of the columns are different, too) of the scatterplot for the ordered pairs of two vectors. As the partitions were not equal, there are many possible partitions with *x* rows and columns of the scatterplot, let *I*_*g*_ denote the MI for one possible partition with *x* × *y* grids that are applied to the ordered samples of the two vectors. For fair comparison between grids of different resolution, the *MI* values of different partitions with *x* × *y* grids of scatterplot for the ordered pairs of two vectors are normalized to the interval [0, 1]. The *m*_*x* × *y*_ is defined as

(2) $$m_{x\times y}=\underset{g\epsilon G_{x\times y}}{\max}\{I_{g}\}/\log\min\{x,y\}$$

MIC is the maximal value of *m*_*x* × *y*_ over the ordered pairs (*x, y*), *x* ≤ *n, y* ≤ *n, n* is the length of the vectors (i.e. number of factors in the vector). In practice, to accelerate computation, it is not necessary to compute *m*_*x* × *y*_ over all (*x, y*), *x* = *n, y* = *n*. Alternatively, we can compute the *m*_*x* × *y*_ over all (*x, y*) *xy* < *B*. Empirically, *B* is defined as *B* = *n*^0.6^. We can then define

(3) $$\text{MIC}=\underset{xy\;<\;B}{\max}\{m_{x\times y}\}$$

Additionally, the eMIC can be defined as

(4) $$\text{eMIC}=\text{MIC}-\text{HSIC}=\text{MIC}-{\text{PCC}}^{2}$$

Refer to (Song et al., 2007; Reshef et al., 2011) for more details of the MIC and HSIC.

#### Identification of features with high discriminative power

Due to the noise, registration error, and inter-individual anatomical differences, only a small number of the 6670 features are highly discriminative. To achieve good discriminative performance as well as resistance to noise and individual disparity, the first step of constructing the classification model was selecting those features with high discriminative power to construct the feature space for classification. The discriminative power of a feature can be quantitatively measured by its relevance to classification (Guyon and Elisseeff, 2003). Here, we used the Kendall tau rank correlation coefficient (Kendall and Jean, 1990) which provides a distribution free test of independence between two variables. Let (*x*_1_, *y*_1_), (*x*_2_, *y*_2_),…, (*x*_*N*_, *y*_*N*_) be a set of samples of the joint random variables *X* and *Y* respectively. Any pair of samples (*x*_*i*_, *y*_*i*_) and (*x*_*j*_, *y*_*j*_) are said to be concordant if the ranks for both elements agree. The Kendall tau coefficient is defined as:

(5) $$\tau =\frac{\begin{array}{c} \left(\text{number of concordant sample pairs}\right)-\\ \left(\text{number of discordant sample pairs}\right) \end{array}}{\text{number of total sample pairs}}$$

For any sample (*x*_*i*_, *y*_*i*_), *i* = 1, 2,…, *N* of the variables need only consider the pairs between itself and the rest *N* − 1 samples. When *i* was changed from 1 to *N*, each pairs was counted twice. Then, number of total simple pairs = 0.5 × *N* × (*N* − 1).

Suppose there are *m* samples in the control group and *n* samples in the patient group. Let *x*_*ij*_ denote the functional connectivity feature *i* of the *j*th samples and *y*_*j*_ denote the class label of this sample (+1 for controls and −1 for patients). The Kendall tau correlation coefficient of the functional connectivity feature *i* can be defined as

(6) $$\tau_{i}=\frac{n_{c}-n_{d}}{m\times n}$$

Because the samples in the same groups are neither concordant pairs nor discordant pairs, the relationship between two samples that belong to the same group is not considered, number of total simple pairs = (*N* − *m*) × (*N* − *n*), *N* = *m* + *n*. The *n*_*c*_ and *n*_*d*_ are the numbers of concordant and discordant pairs between the two groups, respectively. For a pair of two-observation data sets {*x*_*ij*_, *y*_*j*_} and {*x*_*ik*_, *y*_*k*_}, it is neither concordant pair nor discordant pair if *y*_*i*_ = *y*_*k*_; it is a concordant pair when

(7) $$\text{sgn}(x_{ij}-x_{ik})=\text{sgn}(y_{i}-y_{k})$$

where sgn( ) is a signum function. Correspondingly, it is a discordant pair when

(8) $$\text{sgn}(x_{ij}-x_{ik})=-\text{sgn}(y_{i}-y_{k})$$

Thus, a positive correlation coefficient τ_*i*_ represents that the *i*th functional connectivity exhibits significant decrease in the patient group compared to the control group, while a negative τ_*i*_ represents that the *i*th functional connectivity increases in the patient group. The discriminative power was defined as the absolute value of the Kendall tau correlation coefficient. Then we ranked all the features according to their discriminative power and selected those with correlation coefficient over a threshold as the final feature set for classification.

Since a leave-one-out cross-validation strategy was introduced to estimate the generalization ability of the classifiers (see below) and the training data set for feature ranking is slightly different in each iteration of the cross-validation, the first selected features differed slightly from iteration to iteration. Therefore, the contribution of different regions to classification was not evenly distributed, and some regions formed many highly discriminating functional connections with other regions, while some did not. Consensus functional connectivity was introduced here, which was defined as the functional connectivity feature appearing in each cross-validation iteration. Region weight, representing the relative contribution to the identification of schizophrenic patients, was denoted by its occurrence number in the consensus functional connections in this study. The consensus functional connectivity discriminative power was denoted by the mean of its discriminative powers across all iterations of the cross-validation.

#### Support vector classification and performance evaluation

When the data set of features with high discriminative power was obtained, support vector machines (SVM) with linear kernel function were employed to solve the classification problem (Vapnik, 1995; Bishop, 2006). Due to the classification results were influenced by the number of involved features, classification accuracies with fixed parameter setting of SVM (LIBSVM3.11: Linear kernel, C = 1) using a wide range of feature number were reported. Due to our limited number of samples, we used a leave-one-out cross-validation strategy to estimate the generalization ability of our classifier. The performance of a classifier can be quantified using the generalization rate (GR, i.e., the rate of all the subjects correctly classified), sensitivity (SS, i.e., the rate of the patients correctly classified) and specificity (SC, i.e., the rate of the controls correctly classified) based on the results of cross-validation.

## Results

We constructed whole brain functional connectivity using the PCC, MIC, and eMIC based on fMRI data collected from schizophrenic patients and matched healthy controls. Multivariate pattern classification was then introduced to determine the discriminative abilities of the three kinds of functional connectivity. Finally, we analyzed the abnormalities of non-linear and linear functional connections in schizophrenia and determined the spatial distribution of the brain regions related to symptoms of schizophrenia.

### Classification

**Figure 1:**
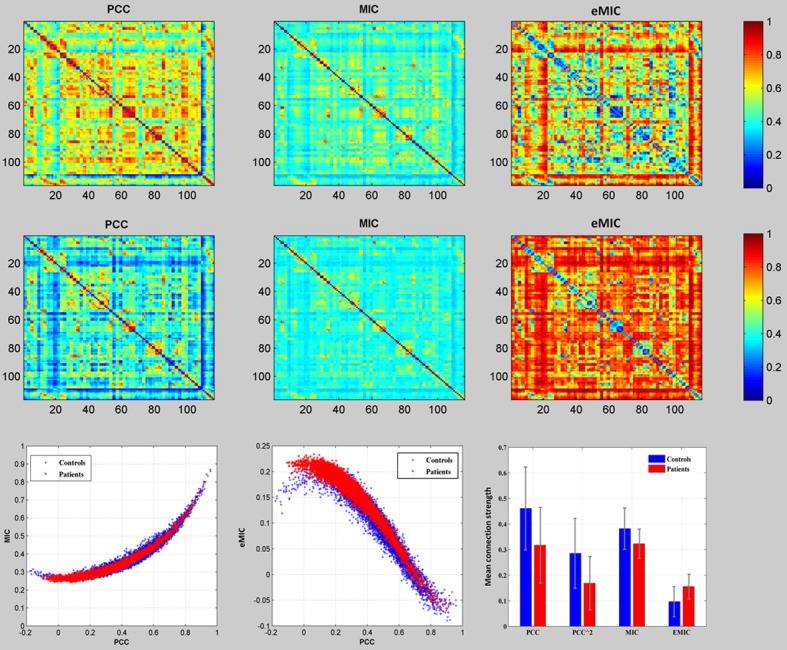
**Comparison of mean functional connectivity of the PCC, MIC, and eMIC**. The **top row** showed the mean functional connectivity maps of the healthy controls, the **mid row** showed the mean functional connectivity maps of the schizophrenic patients, the **bottom row** (the first two) showed the 6670 mean functional connections of the patients (red star) and that of the healthy controls (blue cross) according to MIC, and eMIC against that of the PCC, and (the last one) the comparison of the mean and the deviation of all the 6670 connections according to PCC, MIC, and eMIC between the patients (red bar) and the controls (blue bar).

We examined the whole brain resting-sate functional connectivity of the schizophrenic patients and the healthy controls using PCC, MIC, and eMIC, respectively. The 6670 mean functional connections of the controls (Figure 1, the first row) and the patients (Figure 1, the mid row) according to the PCC, MIC, and eMIC. In the third row of Figure 1, the first two panels were presented the relationship between the MIC and PCC, and between the eMIC and PCC, where each red star represented one of the 6670 mean functional connections over the patients, each blue cross represented one of the 6670 mean functional connections over the controls. The last panel in the third row of Figure 1 showed the mean and deviation of the 6670 mean function connections of the patients (red bar) and the controls (blue bar) which were depicted in the first two panels of the third row. Linear SVM was employed to differentiate the patients with schizophrenia from healthy controls using the whole brain functional connectivity extracted by the PCC, MIC, and eMIC.

**Figure 2:**
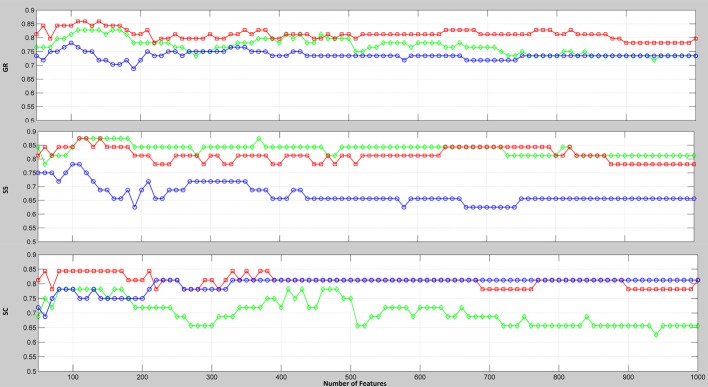
**Classification accuracy rates relative to the number of selected connections extracted by the PCC, MIC, and eMIC**. The *x*-axis represents the number of connections involved in classification; the *y*-axis represents the classification accuracy. GR (the rate of all the subjects correctly classified), SS (the rate of the patients correctly classified), and SC (the rate of the controls correctly classified) are all plotted. Colors represent the types of connection.

To evaluate the discriminative power of the PCC, MIC, and eMIC, we conducted multivariate classification. When the numbers of connections used for classification changed, the classification accuracies changed accordingly. The subjects were classified by using the first connections, and 6670 accuracy rates were obtained. With this approach, the eMIC exhibited excellent performance and the highest classification accuracy rate among the three methods. When the number of features for classification is fixed, the obtained accuracy cannot fully reflect the discriminative power of the whole feature space. Here, we present the classification results using a wide range (50, 100, 150, …, 1000) of the first selected connections (Figure 2). Clearly, the classification accuracies (GR) of the eMIC were consistently higher than the accuracies of the other two methods across the full feature space.

### Changes of functional connectivity

**Figure 3:**
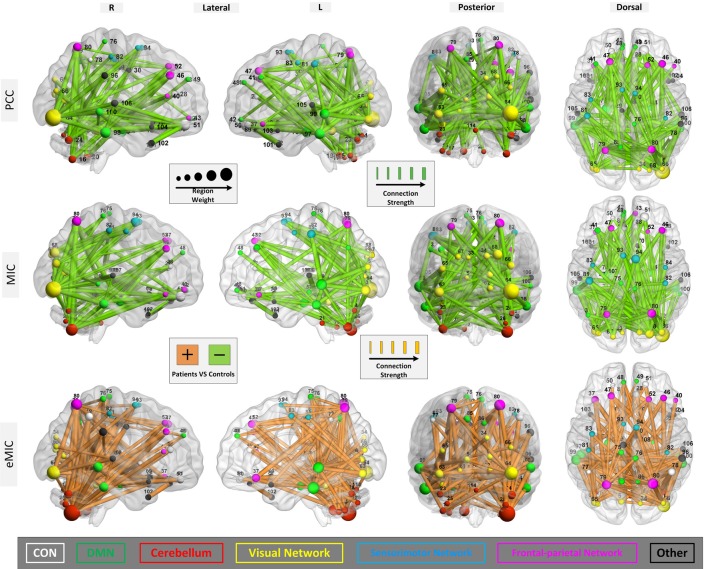
**Region weights and strength of the connections constructed with PCC, MIC, and eMIC**. The connections are displayed in a surface rendering of a human brain. The thicknesses of the consensus connections during leave-one-out cross-validation are scaled by their strength (normalized mean of the rank orders in all iterations during the leave-one-out cross-validation). Connections whose strength were increased in the patients relative to the controls are shown in orange, and connections whose strength were decreased in patients are shown in light green. The ROIs related to the selected consensus connections are also scaled by their weights (sum of the weights of all connections to and from that ROI) and displayed. The ROIs are color-coded according to the functional networks (CON, white; DMN, green; cerebellum, red; visual network, yellow; sensorimotor network, cyan; frontal-parietal network, rose; and other, black). The number in this figure for ROIs is shown in Table A1.

From Figure 2, we can see that when no more than 150 features were involved in the classification, the PCC, MIC, and eMIC gave the best performance. Then, if more features were involved, the classification accuracy of the three methods decreased and the classification accuracy tend to be stable when the number over 200. Therefore, consensus functional connections of the first 200 features involved in the classification corresponding to the PCC, MIC, and eMIC were evaluated. Additionally, the number of features was identified in accordance with previous study (Dosenbach et al., 2010). Then, 113 consensus functional connections were obtained from each iteration of the leave-one-out cross-validation for the MIC and PCC, and 122 consensus functional connections were identified for the eMIC. The consensus functional connections from the PCC, MIC, and eMIC were projected to a surface rendering of a human brain that was visualized with BrainNetViewer (http://www.nitrc.org/projects/bnv/) (see Figure 3). Further, we added a probabilistic atlas of the cerebellum to the ICBM152 cerebrum template that was released with the software.

**Figure 4:**
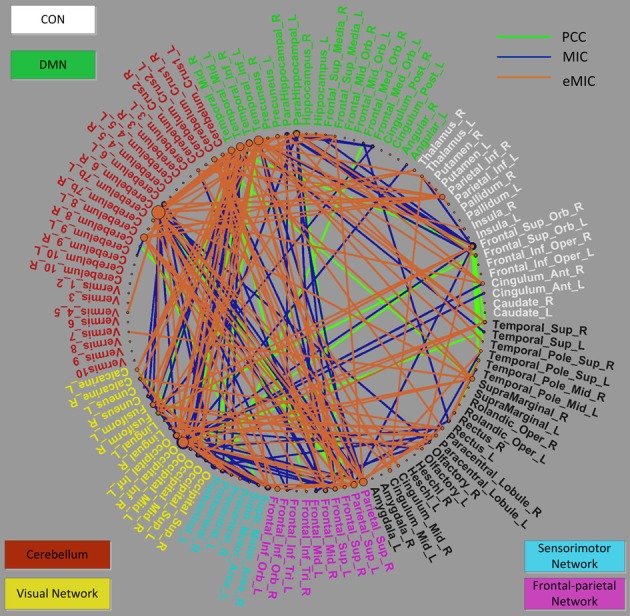
**Distribution of selected consensus connections constructed by the PCC, MIC, and eMIC and region weights of related ROIs demonstrated in a circle graph**. The names of the ROIs are color-coded as shown in Figure 3. The green lines represent connections constructed by the PCC, blue lines represent connections constructed by the MIC, and orange lines represent connections constructed by the eMIC. This figure was plotted using the MATLAB toolbox called PlotPie which was developed by our study group and will be released in the near future.

Primary consideration was given to changes in the strength of the connections. The consensus functional connections according to PCC and MIC were all decreased in patients with schizophrenia compared to healthy controls. It is generally accepted that the strength of functional connectivity is decreased in patients with schizophrenia compared with healthy controls. The PCC- and MIC-based functional connections found in our study are consistent with this common view. In contrast, the strength of the eMIC connections were elevated in patients with schizophrenia. In the Discussion section, we provide a detailed explanation. Additionally, in terms of anatomical location, both the consensus MIC connections and the consensus eMIC connections were largely consistent with the consensus PCC connections. The MIC and the PCC shared 56 common connections, the eMIC and the PCC shared 53 common connections, and the common connections make up approximately half of the total consensus connections. Significant difference of eMIC to PCC is related to the functional connections between the cerebellum and the temporal cortex located in the default model network (DMN) that were found by eMIC but were not detected by PCC. Although there were no PCC functional connections between the cerebellum and the temporal cortex, the cerebellum and the temporal cortex were both connected with the parietal cortex (Figure 4).

### Distribution of functional connectivity

In addition, we investigated the distribution of brain regions that are related to the consensus connections produced by the PCC, MIC, and eMIC. Overall, the distributions of ROIs identified by the PCC, MIC, and eMIC were similar, although the consensus connections were not completely identical across the three methods (Figures 3, 4).

To facilitate analysis, these ROIs can be categorized as follows: (i) the cingulo-opercular network (CON), including several regions in the anterior prefrontal cortex, the inferior parietal cortex, the basal ganglion, the dorsal anterior cingulate cortex (dACC), the insular, the thalamus, and the cerebellum (which will be discussed later separately due to its importance), which is a newly defined cognitive network with great significance for schizophrenia (Dosenbach et al., 2007; Tu et al., 2012); (ii) the DMN, including structures of the hippocampus, the posterior cingulate cortex, the medial prefrontal cortex (mPFC), and the bilateral inferior parietal cortex, which is believed to play an important role in the pathogenesis of schizophrenia (Raichle et al., 2001; Fransson, 2005; Whitfield-Gabrieli et al., 2009); (iii) the cerebellum network, which can be seen as part of the CON; (iv) the visual network comprising the primary visual cortex, the extra-striate visual areas and the lingual gyrus, fusiform gyrus, and calcarine gyrus, which is involved in visual processing (Beckmann and Smith, 2005; Damoiseaux et al., 2006; van den Heuvel et al., 2008; van den Heuvel and Hulshoff Pol, 2010a,b); (v) the sensorimotor network, including the primary sensory cortex, the primary motor cortex the supplementary motor cortex (Biswal et al., 1995; van den Heuvel and Hulshoff Pol, 2010a,b); and (vi) the frontal-parietal network, consisting of the superior parietal and the superior frontal cortex, which is involved in attention processing (Dosenbach et al., 2010; Beckmann et al., 2005; De Luca et al., 2006) (Figure 4).

For the three kinds of functional connectivity, the ROIs with the heaviest weight were distributed primarily in the DMN, cerebellum, visual network, and frontal-parietal network. Specifically, the cerebellum was the most important network according to eMIC, while the visual cortex was the most weighted network according to PCC and MIC.

## Discussion

In this study, we introduced a novel measure called eMIC for estimating the non-linear functional connectivity underlying schizophrenia and applied this estimation of functional connectivity to distinguish schizophrenic patients from healthy controls. Then, we found that strength of the non-linear functional connectivity increased in patients with schizophrenia which was opposed to that observed for the traditional method, which can be attributed to the compensatory mechanisms in the human brain. Furthermore, the non-linear and linear functional connectivity presented similar but not completely the same spatial distribution.

### Analysis of classification

The results of the classifications produced by the PCC, MIC, and eMIC were obtained using the same procedure and the same classifier parameters. The only factor affecting the classification accuracy is the measure of the functional connectivity. Using support vector classification, we compared the discriminative powers of the three kinds of functional connectivity produced by PCC, MIC, and eMIC. The eMIC produced consistently higher classification accuracies than the other two methods across the full connection space (Figure 2). Furthermore, when all 6670 features were used for classification, we obtained the classification results displayed in Table 2. The classification accuracy of the eMIC remained higher than those of the other methods. In short, the functional connectivity produced by the eMIC had the greatest discriminative ability among all three methods.

**Table 2:** **Classification results when all 6670 features were involved**.

**Method (%)**	**SC (%)**	**SS (%)**	**GR (%)**
MIC	71.9	81.2	76.6
PCC	81.2	81.2	81.2
eMIC	81.2	84.4	82.8

Now, we pay more attention to the reason for the better classification accuracy of eMIC. The MIC maximizes the association between two time series, whereas the PCC captures only linear function. Then, the eMIC may capture the subtle non-linear neuronal synchronization in human brain, which will improve the performance of eMIC. From another point of view, the eMIC combines the discriminative information of both the PCC and MIC, which reflects a feature-level information fusion that increased classification accuracy. An MI-based study reported that the linear functions accounted for most of the associations between the fMRI time series and that non-linear functions only mined 5% more information in fMRI time series (Hlinka et al., 2011); this could be explain why the eMIC gave limited improvement to PCC (Figure 2). Conservatively, this result confirmed that the non-linear associations have discriminative power which should not be overlooked. We believe that these non-linear connections add new discriminative information to the linear connections which may increase the classification accuracy.

### Changes of functional connectivity in schizophrenia

Here, we give a detailed analysis of the increased strength of the selected eMIC functional connections. The decreased strength of the PCC functional connections in schizophrenia have been noted in an overwhelming majority of studies (Camchong et al., 2011; Pettersson-Yeo et al., 2011; Repovs et al., 2011; Fornito et al., 2012), which was also confirmed by our previous study (Shen et al., 2010). However, the eMIC-based functional connections demonstrated increased strength in schizophrenic patients compared to healthy controls. As MIC captures various associations but the majority of associations were linear which can be estimated by PCC (Hlinka et al., 2011), decrease of the PCC strength leads to decrease of the MIC strength. The human brain is an organ with great plasticity and adaptability. Thus, the compensatory mechanism of the human brain, that's non-linear correlation will strengthened to reconcile the influence of the corrupt of linear correlation, can result in increases of non-linear functional connectivity. Although the connection selecting procedures of the three methods were implemented separately, the consensus functional connections by the eMIC and the PCC still had a relatively high identity (approximately 50%), which support the notion that the compensatory mechanism of the human brain results in the increase strength of the eMIC in patients with schizophrenia.

If the strength of MIC did not change between the patients and the controls, while the PCC decreased, the increase of the strength of eMIC will be purely caused by the changes of PCC. In fact, the connection strength of MIC decreased in the patients (Figure 1) and gave classification accuracy over 80% (Figure 2). Thus, the increase of eMIC in schizophrenic patients was not simply caused by the decrease of PCC but by the decrease of both PCC and MIC. We believe that the eMIC captures subtle changes of functional connectivity and adds new discriminative information to the classification.

### Network analysis of non-linear functional connectivity

As the eMIC provided useful discriminative information to the linear functional connectivity, aberrant non-linear and linear functional connections were both categorized into six networks for explaining the symptom of schizophrenia. Therein, connections between the DMN and the cerebellum, the DMN and the visual network, the DMN and the frontal-parietal network, and the cerebellum and the frontal-parietal network demonstrated the greatest discriminative power for both the linear and non-linear functional connectivity.

The DMN, the CON (cerebellum), visual network, and frontal-parietal network which are related to specific brain functions were all weighted by the linear and non-linear measures. The DMN was frequently reported in previous studies and weighted in our study. The three methods all identified the important nodes in the DMN such as the mPFC, inferior parietal cortex, Para hippocampal gyrus, and middle temporal cortex. This network is generally accepted as an important network associated with schizophrenia (Bluhm et al., 2007; Garrity et al., 2007; Whitfield-Gabrieli et al., 2009). The CON is believed to support the “task model” in opposition to the “default model” (Dosenbach et al., 2006). In our study, the cerebellum_6_R which can be seen as part of the CON is important for all the three functional connectivity, particularly for the new non-linear measure. Additionally, the PCC, MIC, and eMIC all gave the visual network heavy weight. The frontal-parietal network was also identified by the all the three measures, which was reported as important brain regions in previous studies (Honey et al., 2005; Lynall et al., 2010).

The DMN, the CON (including cerebellum), visual network, and frontal-parietal network obtained slightly different region weight according to the PCC, MIC, and eMIC, respectively. Functional connections between the DMN and the cerebellum were identified by the eMIC but not by the PCC. If the cerebellum is viewed as part of the CON, interactions between the DMN and the CON can be established by the eMIC. The wide spread changes in the CON and DMN may interact with each other, and contribute to the functional basis of schizophrenia. In the connections produced by the PCC and MIC, the right inferior occipital cortex in the visual network exhibited the greatest region weights. However, the cerebellum_6_R exhibited the greatest region weight for the eMIC. This result implies that the cerebellum may play a role in non-linear interaction between different brain regions. Connections between the visual network and regions of the frontal cortex are believed to be involved in visual perception which may contribute to the aberrance of visual perception in schizophrenia (Harvey et al., 2011; Calderone et al., 2013). The cerebellum and the DMN are linked to the frontal-parietal network by eMIC and MIC but not PCC. Combined with the DMN, this network is thought to be closely related to attention tasks (Bush and Shin, 2006; Gao and Lin, 2012), which may explain the occurrence of attention impairment and its relationship to the DMN in schizophrenia.

In conclusion, from the functional network perspective, the distribution of ROIs with the greatest weight according to the linear and non-linear connections was similar but not completely the same, and the non-linear connections shed new light on interpretation of the schizophrenic symptoms.

### Limitations

Although the new functional connectivity constructed by using the eMIC method exhibits better performance in the classification and has explored new information about schizophrenia, there are several limitation in this study. First, our sample size was small. Generalizations of the proposed methods need to be tested with large data sets. Second, the number of slices in the fMRI image used in this study was 20, which was relatively fewer and may not sufficiently to capture the details of the abnormalities in the patients. Third, network analysis of the human brain is a trend in the literature which include ROI localization and connectivity estimation. Our study is confined to connectivity analyses that do not include definition of ROIs.

## Conclusions

In this study, we introduced a novel non-linear functional connectivity for schizophrenia study. The classification results show that the non-linear functional connectivity has an equal if not better discriminative ability than existing linear measures in schizophrenia identification. This result suggests that non-linear functional connectivity should be taken into account in research on schizophrenia and other psychiatric disorders. Furthermore, we found that the non-linear functional connectivity which was strengthened in the patients has a similar distribution with its linear counterpart. This new finding indicates the presence of compensatory mechanism between linear and non-linear associations and non-linear functional network abnormalities underlying the symptom of schizophrenia.

## Study funding

This study was supported by the National Basic Research Program of China (2011CB707802) and the National Natural Science Foundation of China (61003202, 61005084).

### Conflict of interest statement

The authors declare that the research was conducted in the absence of any commercial or financial relationships that could be construed as a potential conflict of interest.

## Appendix

**Table A1:** **Names of the ROIs used in this study**.

1. Amygdala_L
2. Amygdala_R
3. Angular_L
4. Angular_R
5. Calcarine_L
6. Calcarine_R
7. Caudate_L
8. Caudate_R
9. Cerebelum_3_L
10. Cerebelum_3_R
11. Cerebelum_4_5_L
12. Cerebelum_4_5_R
13. Cerebelum_6_L
14. Cerebelum_6_R
15. Cerebelum_7b_L
16. Cerebelum_7b_R
17. Cerebelum_8_L
18. Cerebelum_8_R
19. Cerebelum_9_L
20. Cerebelum_9_R
21. Cerebelum_10_L
22. Cerebelum_10_R
23. Cerebelum_Crus1_L
24. Cerebelum_Crus1_R
25. Cerebelum_Crus2_L
26. Cerebelum_Crus2_R
27. Cingulum_Ant_L
28. Cingulum_Ant_R
29. Cingulum_Mid_L
30. Cingulum_Mid_R
31. Cingulum_Post_L
32. Cingulum_Post_R
33. Cuneus_L
34. Cuneus_R
35. Frontal_Inf_Oper_L
36. Frontal_Inf_Oper_R
37. Frontal_Inf_Orb_L
38. Frontal_Inf_Orb_R
39. Frontal_Inf_Tri_L
40. Frontal_Inf_Tri_R
41. Frontal_Med_Orb_L
42. Frontal_Med_Orb_R
43. Frontal_Mid_L
44. Frontal_Mid_Orb_L
45. Frontal_Mid_Orb_R
46. Frontal_Mid_R
47. Frontal_Sup_L
48. Frontal_Sup_Media_L
49. Frontal_Sup_Media_R
50. Frontal_Sup_Orb_L
51. Frontal_Sup_Orb_R
52. Frontal_Sup_R
53. Fusiform_L
54. Fusiform_R
55. Heschl_L
56. Heschl_R
57. Hippocampus_L
58. Hippocampus_R
59. Insula_L
60. Insula_R
61. Lingual_L
62. Lingual_R
63. Occipital_Inf_L
64. Occipital_Inf_R
65. Occipital_Mid_L
66. Occipital_Mid_R
67. Occipital_Sup_L
68. Occipital_Sup_R
69. Olfactory_L
70. Olfactory_R
71. Pallidum_L
72. Pallidum_R
73. Paracentral_Lobule_L
74. Paracentral_Lobule_R
75. ParaHippocampal_L
76. ParaHippocampal_R
77. Parietal_Inf_L
78. Parietal_Inf_R
79. Parietal_Sup_L
80. Parietal_Sup_R
81. Postcentral_L
82. Postcentral_R
83. Precentral_L
84. Precentral_R
85. Precuneus_L
86. Precuneus_R
87. Putamen_L
88. Putamen_R
89. Rectus_L
90. Rectus_R
91. Rolandic_Oper_L
92. Rolandic_Oper_R
93. Supp_Motor_Area_L
94. Supp_Motor_Area_R
95. SupraMarginal_L
96. SupraMarginal_R
97. Temporal_Inf_L
98. Temporal_Inf_R
99. Temporal_Mid_L
100. Temporal_Mid_R
101. Temporal_Pole_Mid_L
102. Temporal_Pole_Mid_R
103. Temporal_Pole_Sup_L
104. Temporal_Pole_Sup_R
105. Temporal_Sup_L
106. Temporal_Sup_R
107. Thalamus_L
108. Thalamus_R
109. Vermis_1_2
110. Vermis_3
111. Vermis_4_5
112. Vermis_6
113. Vermis_7
114. Vermis_8
115. Vermis_9
116. Vermis10

## References

[B1] Achard S. Salvador R. Whitcher B. Suckling J. Bullmore E. (2006). A resilient, low-frequency, small-world human brain functional network with highly connected association cortical hubs. J. Neurosci. 26, 63–72 10.1523/JNEUROSCI.3874-05.2006 16399673 PMC6674299

[B2] Alexander-Bloch A. F. Vertes P. E. Stidd R. Lalonde F. Clasen L. Rapoport J. (2013). The anatomical distance of functional connections predicts brain network topology in health and schizophrenia. Cereb. Cortex 23, 127–138 10.1093/cercor/bhr388 22275481 PMC3513955

[B3] Andreasen N. C. Paradiso S. O'Leary D. S. (1998). “Cognitive dysmetria” as an integrative theory of schizophrenia: a dysfunction in cortical-subcortical-cerebellar circuitry? Schizophr. Bull. 24, 203–218 10.1093/oxfordjournals.schbul.a033321 9613621

[B4] Bassett D. S. Nelson B. G. Mueller B. A. Camchong J. Lim K. O. (2012). Altered resting state complexity in schizophrenia. Neuroimage 59, 2196–2207 10.1016/j.neuroimage.2011.10.002 22008374 PMC3254701

[B5] Beckmann C. F. DeLuca M. Devlin J. T. Smith S. M. (2005). Investigations into resting-state connectivity using independent component analysis. Philos. Trans. R. Soc. Lond. B Biol. Sci. 360, 1001–1013 10.1098/rstb.2005.1634 16087444 PMC1854918

[B6] Beckmann C. F. Smith S. M. (2005). Tensorial extensions of independent component analysis for multisubject FMRI analysis. Neuroimage 25, 294–311 10.1016/j.neuroimage.2004.10.043 15734364

[B7] Bishop C. M. (2006). Pattern Recognition and Machine Learning. Singapore: Springer

[B8] Biswal B. DeYoe A. E. Hyde J. S. (1996). Reduction of physiological fluctuations in fMRI using digital filters. Magn. Reson. Med. 35, 107–113 10.1002/mrm.1910350114 8771028

[B9] Biswal B. Yetkin F. Z. Haughton V. M. Hyde J. S. (1995). Functional connectivity in the motor cortex of resting human brain using echo-planar MRI. Magn. Reson. Med. 34, 537–541 10.1002/mrm.1910340409 8524021

[B10] Bluhm R. L. Miller J. Lanius R. A. Osuch E. A. Boksman K. Neufeld R. W. (2007). Spontaneous low-frequency fluctuations in the BOLD signal in schizophrenic patients: anomalies in the default network. Schizophr. Bull. 33, 1004–1012 10.1093/schbul/sbm052 17556752 PMC2632312

[B11] Buckner R. L. Koutstaal W. Schacter D. L. Dale A. M. Rotte M. Rosen B. R. (1998). Functional-anatomic study of episodic retrieval. II. Selective averaging of event-related fMRI trials to test the retrieval success hypothesis. Neuroimage 7, 163–175 10.1006/nimg.1998.0328 9597658

[B12] Bush G. Shin L. M. (2006). The Multi-Source Interference Task: an fMRI task that reliably activates the cingulo-frontal-parietal cognitive/attention network. Nat. Protoc. 1, 308–313 10.1038/nprot.2006.48 17406250

[B13] Buxton R. B. Wong E. C. Frank L. R. (1998). Dynamics of blood flow and oxygenation changes during brain activation: the balloon model. Magn. Reson. Med. 39, 855–864 10.1002/mrm.1910390602 9621908

[B14] Calderone D. J. Hoptman M. J. Martínez A. Nair-Collins S. Mauro C. J. Bar M. (2013). Contributions of low and high spatial frequency processing to impaired object recognition circuitry in schizophrenia. Cereb. Cortex 23, 1849–1858 10.1093/cercor/bhs169 22735157 PMC3698366

[B15] Camchong J. MacDonald A. W. 3rd. Bell C. Mueller B. A. Lim K. O. (2011). Altered functional and anatomical connectivity in schizophrenia. Schizophr. Bull. 37, 640–650 10.1093/schbul/sbp131 19920062 PMC3080691

[B16] Cecchi G. A. Rao A. R. Centeno M. V. Baliki M. Apkarian A. V. Chialvo D. R. (2007). Identifying directed links in large scale functional networks: application to brain fMRI. BMC Cell Biol. 8 Suppl. 1:S5 10.1186/1471-2121-8-S1-S5 17634095 PMC1924510

[B17] Cordes D. Haughton V. M. Arfanakis K. Wendt G. J. Turski P. A. Moritz C. H. (2000). Mapping functionally related regions of brain with functional connectivity MR imaging. Am. J. Neuroradiol. 21, 1636–1644 11039342 PMC8174861

[B18] Damoiseaux J. S. Rombouts S. A. R. B. Barkhof F. Scheltens P. Stam C. J. Smith S. M. (2006). Consistent resting-state networks across healthy subjects. Proc. Natl. Acad. Sci. U.S.A. 103, 13848–13853 10.1073/pnas.0601417103 16945915 PMC1564249

[B19] Daunizeau J. Stephan K. E. Friston K. J. (2012). Stochastic dynamic causal modelling of fMRI data: should we care about neural noise? Neuroimage 62, 464–481 10.1016/j.neuroimage.2012.04.061 22579726 PMC3778887

[B20] De Luca M. Beckmann C. F. De Stefano N. Matthews P. M. Smith S. M. (2006). fMRI resting state networks define distinct modes of long-distance interactions in the human brain. Neuroimage 29, 1359–1367 10.1016/j.neuroimage.2005.08.035 16260155

[B21] de Zwart J. A. van Gelderen P. Jansma J. M. Fukunaga M. Bianciardi M. Duyn J. H. (2009). Hemodynamic nonlinearities affect BOLD fMRI response timing and amplitude. Neuroimage 47, 1649–1658 10.1016/j.neuroimage.2009.06.001 19520175 PMC2731556

[B22] Dosenbach N. U. Nardos B. Cohen A. L. Fair D. A. Power J. D. Church J. A. (2010). Prediction of individual brain maturity using fMRI. Science 329, 1358–1361 10.1126/science.1194144 20829489 PMC3135376

[B23] Dosenbach N. U. F. Fair D. A. Miezin F. M. Cohen A. L. Wenger K. K. Dosenbach R. A. T. (2007). Distinct brain networks for adaptive and stable task control in humans. Proc. Natl. Acad. Sci. U.S.A. 104, 11073–11078 10.1073/pnas.0704320104 17576922 PMC1904171

[B24] Dosenbach N. U. F. Visscher K. M. Palmer E. D. Miezin F. M. Wenger K. K. Kang H. S. C. (2006). A core system for the implementation of task sets. Neuron 50, 799–812 10.1016/j.neuron.2006.04.031 16731517 PMC3621133

[B25] Fallani F. D. V. Maglione A. Babiloni F. Mattia D. Astolfi L. Vecchiato G. (2010). Cortical network analysis in patients affected by schizophrenia. Brain Topogr. 23, 214–220 10.1007/s10548-010-0133-2 20094766

[B26] Fan Y. Liu Y. Wu H. Hao Y. Liu H. Liu Z. (2011). Discriminant analysis of functional connectivity patterns on Grassmann manifold. Neuroimage 56, 2058–2067 10.1016/j.neuroimage.2011.03.051 21440643

[B27] Fornito A. Zalesky A. Pantelis C. Bullmore E. T. (2012). Schizophrenia, neuroimaging and connectomics. Neuroimage 62, 2296–2314 10.1016/j.neuroimage.2011.12.090 22387165

[B28] Fransson P. (2005). Spontaneous low-frequency BOLD signal fluctuations: an fMRI investigation of the resting-state default mode of brain function hypothesis. Hum. Brain Mapp. 26, 15–29 10.1002/hbm.20113 15852468 PMC6871700

[B29] Friston K. J. Frith C. D. (1995). Schizophrenia: a disconnection syndrome? Clin. Neurosci. 3, 89–97 7583624

[B30] Friston K. J. Frith C. D. Liddle P. F. Frackowiak R. S. (1993). Functional connectivity: the principal-component analysis of large (PET) data sets. J. Cereb. Blood Flow Metab. 13, 5–14 10.1038/jcbfm.1993.4 8417010

[B31] Friston K. J. Harrison L. Penny W. (2003). Dynamic causal modelling. Neuroimage 19, 1273–1302 10.1016/S1053-8119(03)00202-7 12948688

[B32] Gao W. Lin W. (2012). Frontal parietal control network regulates the anti-correlated default and dorsal attention networks. Hum. Brain Mapp. 33, 192–202 10.1002/hbm.21204 21391263 PMC3131466

[B33] Garrity A. G. Pearlson G. D. McKiernan K. Lloyd D. Kiehl K. A. Calhoun V. D. (2007). Aberrant “default mode” functional connectivity in schizophrenia. Am. J. Psychiatry 164, 450–457 10.1176/appi.ajp.164.3.450 17329470

[B34] Guyon I. Elisseeff A. (2003). An introduction to variable and feature selection. J. Mach. Learn. Res. 3, 1157–1182

[B35] Hampson M. Peterson B. S. Skudlarski P. Gatenby J. C. Gore J. C. (2002). Detection of functional connectivity using temporal correlations in MR images. Hum. Brain Mapp. 15, 247–262 10.1002/hbm.10022 11835612 PMC6872035

[B36] Harvey P.-O. Lee J. Cohen M. S. Engel S. A. Glahn D. C. Nuechterlein K. H. (2011). Altered dynamic coupling of lateral occipital complex during visual perception in schizophrenia. Neuroimage 55, 1219–1226 10.1016/j.neuroimage.2010.12.045 21194569 PMC3049854

[B37] Hlinka J. Palus M. Vejmelka M. Mantini D. Corbetta M. (2011). Functional connectivity in resting-state fMRI: is linear correlation sufficient? Neuroimage 54, 2218–2225 10.1016/j.neuroimage.2010.08.042 20800096 PMC4139498

[B38] Honey G. D. Pomarol-Clotet E. Corlett P. R. Honey R. A. McKenna P. J. Bullmore E. T. (2005). Functional dysconnectivity in schizophrenia associated with attentional modulation of motor function. Brain 128(Pt 11), 2597–2611 10.1093/brain/awh632 16183659 PMC3838931

[B39] Insel T. Cuthbert B. Garvey M. Heinssen R. Pine D. S. Quinn K. (2010). Research domain criteria (RDoC): toward a new classification framework for research on mental disorders. Am. J. Psychiatry 167, 748–751 10.1176/appi.ajp.2010.09091379 20595427

[B40] Kendall M. G. Jean D. G. (1990). Rank Correlation Methods. NewYork, NY: Oxford University Press 10.2307/2333282

[B41] Kendler K. S. (2009). An historical framework for psychiatric nosology. Psychol. Med. 39, 1935–1941 10.1017/S0033291709005753 19368761 PMC2783473

[B42] Lahaye P. J. Poline J. B. Flandin G. Dodel S. Garnero L. (2003). Functional connectivity: studying nonlinear, delayed interactions between BOLD signals. Neuroimage 20, 962–974 10.1016/S1053-8119(03)00340-9 14568466

[B43] Lee S. P. Duong T. Q. Yang G. Iadecola C. Kim S. G. (2001). Relative changes of cerebral arterial and venous blood volumes during increased cerebral blood flow: implications for BOLD fMRI. Magn. Reson. Med. 45, 791–800 10.1002/mrm.1107 11323805

[B44] Li K. Guo L. Nie J. Li G. Liu T. (2009). Review of methods for functional brain connectivity detection using fMRI. Comput. Med. Imaging Graph. 33, 131–139 10.1016/j.compmedimag.2008.10.011 19111443 PMC2724763

[B45] Liang M. Zhou Y. Jiang T. Liu Z. Tian L. Liu H. (2006). Widespread functional disconnectivity in schizophrenia with resting-state functional magnetic resonance imaging. Neuroreport 17, 209–213 10.1097/01.wnr.0000198434.06518.b8 16407773

[B46] Liu H. Liu Z. Liang M. Hao Y. Tan L. Kuang F. (2006). Decreased regional homogeneity in schizophrenia: a resting state functional magnetic resonance imaging study. Neuroreport 17, 19–22 10.1097/01.wnr.0000195666.22714.35 16361943

[B47] Liu H. H. Kaneko Y. Ouyang X. Li L. Hao Y. H. Chen E. Y. H. (2012). Schizophrenic patients and their unaffected siblings share increased resting-state connectivity in the task-negative network but not its anticorrelated task-positive network. Schizophr. Bull. 38, 285–294 10.1093/schbul/sbq074 20595202 PMC3283150

[B48] Lowe M. J. Dzemidzic M. Lurito J. T. Mathews V. P. Phillips M. D. (2000). Correlations in low-frequency BOLD fluctuations reflect cortico-cortical connections. Neuroimage 12, 582–587 10.1006/nimg.2000.0654 11034865

[B49] Lowe M. J. Mock B. J. Sorenson J. A. (1998). Functional connectivity in single and multislice echoplanar imaging using resting-state fluctuations. Neuroimage 7, 119–132 10.1006/nimg.1997.0315 9558644

[B50] Lynall M. E. Bassett D. S. Kerwin R. McKenna P. J. Kitzbichler M. Muller U. (2010). Functional connectivity and brain networks in schizophrenia. J. Neurosci. 30, 9477–9487 10.1523/JNEUROSCI.0333-10.2010 20631176 PMC2914251

[B51] Maldjian J. A. Laurienti P. J. Kraft R. A. Burdette J. H. (2003). An automated method for neuroanatomic and cytoarchitectonic atlas-based interrogation of fMRI data sets. Neuroimage 19, 1233–1239 10.1016/S1053-8119(03)00169-1 12880848

[B52] Miezin F. M. Maccotta L. Ollinger J. M. Petersen S. E. Buckner R. L. (2000). Characterizing the hemodynamic response: effects of presentation rate, sampling procedure, and the possibility of ordering brain activity based on relative timing. Neuroimage 11(6 Pt 1), 735–759 10.1006/nimg.2000.0568 10860799

[B53] Pettersson-Yeo W. Allen P. Benetti S. McGuire P. Mechelli A. (2011). Dysconnectivity in schizophrenia: where are we now? Neurosci. Biobehav. Rev. 35, 1110–1124 10.1016/j.neubiorev.2010.11.004 21115039

[B54] Raichle M. E. MacLeod A. M. Snyder A. Z. Powers W. J. Gusnard D. A. Shulman G. L. (2001). A default mode of brain function. Proc. Natl. Acad. Sci. U.S.A. 98, 676–682 10.1073/pnas.98.2.676 11209064 PMC14647

[B55] Repovs G. Csernansky J. G. Barch D. M. (2011). Brain Network Connectivity in Individuals with Schizophrenia and Their Siblings. Biol. Psychiatry 69, 967–973 10.1016/j.biopsych.2010.11.009 21193174 PMC3081915

[B56] Reshef D. N. Reshef Y. A. Finucane H. K. Grossman S. R. McVean G. Turnbaugh P. J. (2011). Detecting novel associations in large data sets. Science 334, 1518–1524 10.1126/science.1205438 22174245 PMC3325791

[B57] Saad Z. S. Ropella K. M. Cox R. W. DeYoe E. A. (2001). Analysis and use of FMRI response delays. Hum. Brain Mapp. 13, 74–93 10.1002/hbm.1026 11346887 PMC6872085

[B58] Salvador R. Sarro S. Gomar J. J. Ortiz-Gil J. Vila F. Capdevila A. (2010). Overall brain connectivity maps show cortico—subcortical abnormalities in schizophrenia. Hum. Brain Mapp. 31, 2003–2014 10.1002/hbm.20993 20225222 PMC6870792

[B59] Schmahmann J. D. Doyon J. McDonald D. Holmes C. Lavoie K. Hurwitz A. S. (1999). Three-dimensional MRI atlas of the human cerebellum in proportional stereotaxic space. Neuroimage 10(3 Pt 1), 233–260 10.1006/nimg.1999.0459 10458940

[B60] Shen H. Wang L. Liu Y. Hu D. (2010). Discriminative analysis of resting-state functional connectivity patterns of schizophrenia using low dimensional embedding of fMRI. Neuroimage 49, 3110–3121 10.1016/j.neuroimage.2009.11.011 19931396

[B61] Smith S. M. Miller K. L. Salimi-Khorshidi G. Webster M. Beckmann C. F. Nichols T. E. (2011). Network modelling methods for FMRI. Neuroimage 54, 875–891 10.1016/j.neuroimage.2010.08.063 20817103

[B62] Song L. Smola A. Gretton A. Borgwardt K. (2007). Supervised Feature selection via dependence estimation, in Machine Learning, ed Ghahramani Z. (NewYork, NY: Omnipress), 823–830 10.1145/1273496.1273600

[B63] Sui J. Yu Q. He H. Pearlson G. D. Calhoun V. D. (2012). A selective review of multimodal fusion methods in schizophrenia. Front. Hum. Neurosci. 6:27 10.3389/fnhum.2012.00027 22375114 PMC3285795

[B64] Sun F. T. Miller L. M. D'Esposito M. (2004). Measuring interregional functional connectivity using coherence and partial coherence analyses of fMRI data. Neuroimage 21, 647–658 10.1016/j.neuroimage.2003.09.056 14980567

[B65] Tu P.-C. Hsieh J.-C. Li C.-T. Bai Y.-M. Su T.-P. (2012). Cortico-striatal disconnection within the cingulo-opercular network in schizophrenia revealed by intrinsic functional connectivity analysis: a resting fMRI study Neuroimage 59, 238–247 10.1016/j.neuroimage.2011.07.086 21840407

[B66] Tzourio-Mazoyer N. Landeau B. Papathanassiou D. Crivello F. Etard O. Delcroix N. (2002). Automated anatomical labeling of activations in SPM using a macroscopic anatomical parcellation of the MNI MRI single-subject brain. Neuroimage 15, 273–289 10.1006/nimg.2001.0978 11771995

[B67] van den Heuvel M. P. Hulshoff Pol H. E. (2010a). Exploring the brain network: a review on resting-state fMRI functional connectivity. Eur. Neuropsychopharmacol. 20, 519–534 10.1016/j.euroneuro.2010.03.008 20471808

[B68] van den Heuvel M. P. Hulshoff Pol H. E. (2010b). Specific somatotopic organization of functional connections of the primary motor network during resting state. Hum. Brain Mapp. 31, 631–644 10.1016/j.euroneuro.2010.03.008 19830684 PMC6871083

[B69] van den Heuvel M. Mandl R. Hulshoff Pol H. (2008). Normalized cut group clustering of resting-state FMRI data. PLoS ONE 3:e2001 10.1371/journal.pone.0002001 18431486 PMC2291558

[B70] van Os J. Kenis G. Rutten B. P. (2010). The environment and schizophrenia. Nature 468, 203–212 10.1038/nature09563 21068828

[B71] Vapnik V. (1995). The Nature of Statistical Learning Theory. NewYork, NY: Springer 10.10007/978-1-4757-2440-0

[B72] Vazquez A. L. Noll D. C. (1998). Nonlinear aspects of the BOLD response in functional MRI. Neuroimage 7, 108–118 10.1006/nimg.1997.0316 9558643

[B73] Vertes P. E. Alexander-Bloch A. F. Gogtay N. Giedd J. N. Rapoport J. L. Bullmore E. T. (2012). Simple models of human brain functional networks. Proc. Natl. Acad. Sci. U.S.A. 109, 5868–5873 10.1073/pnas.1111738109 22467830 PMC3326510

[B74] Whitfield-Gabrieli S. Thermenos H. W. Milanovic S. Tsuang M. T. Faraone S. V. McCarley R. W. (2009). Hyperactivity and hyperconnectivity of the default network in schizophrenia and in first-degree relatives of persons with schizophrenia. Proc. Natl. Acad. Sci. U.S.A. 106, 1279–1284 10.1073/pnas.0809141106 19164577 PMC2633557

[B75] Xie X. Cao Z. Weng X. (2008a). Spatiotemporal nonlinearity in resting-state fMRI of the human brain. Neuroimage 40, 1672–1685 10.1016/j.neuroimage.2008.01.007 18316208

[B76] Xie X. Cao Z. Weng X. (2008b). Detecting spatiotemporal nonlinear dynamics in resting state of human brain based on fMRI datasets. Neuroimage 205, 19–25 10.1016/j.neuroimage.2008.01.007 18316208

[B77] Xiong J. Parsons L. M. Gao J. H. Fox P. T. (1999). Interregional connectivity to primary motor cortex revealed using MRI resting state images. Hum. Brain Mapp. 8, 151–156 10.1002/(SICI)1097-0193(1999)8:2/3<151::AID-HBM13>3.0.CO;2-5 10524607 PMC6873334

[B78] Zalesky A. Fornito A. Egan G. F. Pantelis C. Bullmore E. T. (2012). The relationship between regional and inter-regional functional connectivity deficits in schizophrenia. Hum. Brain Mapp. 33, 2535–2549 10.1002/hbm.21379 21922601 PMC6870162

[B79] Zhang N. Zhu X. H. Chen W. (2008). Investigating the source of BOLD nonlinearity in human visual cortex in response to paired visual stimuli. Neuroimage 43, 204–212 10.1016/j.neuroimage.2008.06.033 18657623 PMC2614294

